# Impact of Seasonal and Temperature-Dependent Variation in Root Defense Metabolites on Herbivore Preference in *Taraxacum officinale*

**DOI:** 10.1007/s10886-019-01126-9

**Published:** 2019-12-12

**Authors:** Wei Huang, Zoe Bont, Maxime R. Hervé, Christelle A. M. Robert, Matthias Erb

**Affiliations:** 1 Institute of Plant Sciences, University of Bern, Altenbergrain 21, 3013 Bern, Switzerland; 2 CAS Key Laboratory of Aquatic Botany and Watershed Ecology, Wuhan Botanical Garden, Chinese Academy of Sciences, Wuhan, 430074 Hubei China; 3 Inra, Agrocampus Ouest, IGEPP – UMR-A 1349, University of Rennes, F-35000 Rennes, France

**Keywords:** Below ground herbivory, Circannual rhythms, *Melolontha melolontha*, Root defense, Primary and secondary metabolites, *Taraxacum officinale*

## Abstract

Plants experience seasonal fluctuations in abiotic and biotic factors such as herbivore attack rates. If and how root defense expression co-varies with seasonal fluctuations in abiotic factors and root herbivore attack rates is not well understood. Here, we evaluated seasonal changes in defensive root latex chemistry of *Taraxacum officinale* plants in the field and correlated the changes with seasonal fluctuations in abiotic factors and damage potential by *Melolontha melolontha*, a major natural enemy of *T. officinale*. We then explored the causality and consequences of these relationships under controlled conditions. The concentration of the defensive sesquiterpene lactone taraxinic acid β-D glucopyranosyl ester (TA-G) varied substantially over the year and was most strongly correlated to mean monthly temperature. Both temperature and TA-G levels were correlated with annual fluctuations in potential *M. melolontha* damage. Under controlled conditions, plants grown under high temperature produced more TA-G and were less attractive for *M. melolontha*. However, temperature-dependent *M. melolontha* feeding preferences were not significantly altered in TA-G deficient transgenic lines. Our results suggest that fluctuations in temperature leads to variation in the production of a root defensive metabolites that co-varies with expected attack of a major root herbivore. Temperature-dependent herbivore preference, however, is likely to be modulated by other phenotypic alterations.

**Electronic supplementary material:**

The online version of this article (10.1007/s10886-019-01126-9) contains supplementary material, which is available to authorized users.

## Introduction

Over the course of the year, plants are exposed to substantial environmental variation, including diurnal and seasonal fluctuations in temperature, precipitation, photoperiod, pest and pathogen pressure (McClung 2006; Poelman et al. 2008; Sanchez et al. 2011). Synchronizing the production of resistance factors such as toxic secondary metabolites with the occurrence of biotic threats against a background of abiotic fluctuations is therefore a major challenge for plants (Ingle et al. 2015; Wang et al. 2011). Plants may overcome this challenge for instance by inducing defenses independently of abiotic factors or by using abiotic factors as predictors of natural enemy attack (Greenham and McClung 2015; Sanchez and Kay 2016).

Over the last years, substantial progress has been made in understanding the role of diurnal variation in plant-herbivore and plant-pathogen interactions (Baldwin and Meldau 2013; Seo and Mas 2015; Sharma and Bhatt 2015). The emerging picture is that diurnal variation in abiotic entrains the circadian clock (McClung 2006; Sanchez et al. 2011), and this entrainment regulates defenses, leading in some cases to the highest defense expression at times when pathogen attack is also most likely to occur (Wang et al. 2011; Zhang et al. 2013; Zhou et al. 2015). In addition, plants often respond strongly to pathogen and herbivore attack and thereby gain control over defense deployment (Dodds and Rathjen 2010; Karban 2011). Although defense induction is modulated by diurnal variation in abiotic factors and the circadian clock, this variation is often lower than the magnitude of induced responses (Arimura et al. 2008; Herden et al. 2016; McCormick et al. 2014). Thus, plant defenses are modulated by both abiotic entrainment and independent regulation across the day.

In contrast to diurnal patterns, less is known about the synchronization of plant defenses to seasonal variation. Similar to the circadian clock, perennial plants can entrain their circannual clock using zeitgebers such as photoperiod and temperature and then regulate their development to anticipate seasonal changes (Gwinner 1986). Several studies document that the accumulation of plant secondary metabolites varies substantially across seasons (Bowers et al. 1992; Carvalho et al. 2014; Feeny 1970; Hare 2010; Liimatainen et al. 2012). However, different defense metabolites display different seasonal patterns. For example, Riipi et al. (2002) found that concentration of soluble proanthocyanidins in the leaf of mountain birch increased linearly throughout the season, while cell wall-bound proanthocyanidins exhibited unimodal relationship with season. Solar et al. (2006) reported that flavonoids in the shoot of common walnut increased from the spring to the summer, while phenolic acids showed an opposite pattern, with highest concentrations in spring and lowest concentrations in the summer. Gols et al. (2018) also showed seasonal dynamics of defense metabolites in the leaf of cabbage that aliphatic glucosinolates gradually increased with growing season, while indole glucosinolates rapidly increased until mid-summer and then decreased or stabilized. To what extent these seasonal fluctuations depend on environmental factors such as temperature, precipitation and photoperiod, and to what extent they are synchronized with natural enemy attack as a form of optimal defense remains poorly understood (Chittka and Döring 2007). Many of the investigated metabolites may also help plants to cope with abiotic stress (Hartmann 2007; Vaughan et al. 2015). Seasonal fluctuations may therefore also reflect these additional functions. Alternatively, seasonal fluctuations may be the result of physiological constraint imposed by abiotic stress.

Seasonal fluctuations in plant defenses co-occur with many other phenotypic alterations that may affect plant attractiveness and resistance to herbivores. Concentrations of plant primary metabolites, including amino acids and sugars, for instance, vary substantially during growing season (Budzinski et al. 2016; Riipi et al. 2002) and are important determinants of plant quality for herbivores (Behmer 2009; Erb et al. 2013). Moreover, seasonal changes in water supply can alter physiological characteristics such as turgor pressure (Mitchell et al. 2008; Simpson et al. 2012) and water content (Claussen 2005; Fernàndez-Martínez et al. 2013) and, thereby, influence plant palatability (Huberty and Denno 2004). In addition, traits related to structural defenses including tissue toughness and cuticle thickness have been reported to vary across seasons (Gotsch et al. 2010) and may contribute to circannual patterns of herbivore resistance (Peeters 2002; Uyi et al. 2018). Hence, plant resistance to herbivores and herbivore performance may be influenced by a wide variety of plant traits apart from plant defense metabolites (Hu et al. 2018; Huang et al. 2019). So far, the relative contribution of plant defense metabolites to seasonal fluctuations in plant resistance to herbivores is poorly understood.

Seasonal fluctuations in plant defense expression have so far mostly been studied above ground, and little is known about root defenses in this respect. Nelson et al. (1981) analyzed the cardenolides in the root of milkweed for 1 year, but did not find any significant change throughout the season. Robakowski et al. (2016) found that season had a pronounced effect on root prunasin levels, which were low in May, increased until mid-summer and then decreased again. Given that belowground herbivores are ubiquitous in natural systems, and that many of them spend several years in the soil feeding on plants as immatures before emerging as adults (van Dam 2009), understanding how root defenses vary across seasons and if this variation is associated with increased resistance and the probability of attack is an open question in the field of below ground plant-herbivore interactions.

To understand if and how seasonal variation influences plant defense metabolites in the roots, if this fluctuation is associated with expected herbivore attack rates, and to what extent this fluctuation can explain herbivore preferences, we studied these processes in the common dandelion (*Taraxacum officinale*, Asteraceae). *T. officinale* is a perennial flowering herb with a wide distribution across Eurasia. One of the major pests of *T. officinale* is the European cockchafer (*Melolontha melolontha*, Coleoptera; Scarabaeidae) (Huber et al. 2016a). Larvae of *M. melolontha* prefer to feed on the roots of *T. officinale* and cause serious damage from mid-April to October (Hauss and Schütte 1976, 1978). *M. melolontha* larvae typically overwinter in deeper soil layers and move up to feed on plant roots around mid-April, with feeding and damage peaking in July and August (Spinatsch 2010). Other root herbivores that are less damaging to *T. officinale* such as wireworms show different seasonal patterns (Jung et al. 2012). The roots of *T. officinale* produce high amounts of bitter latex that mainly consists of phenolic inositol esters with two 4-hydroxyphenylacetic acid side groups (Di-PIEs), phenolic inositol esters with three 4-hydroxyphenylacetic acid side groups (Tri-PIEs), triterpene acetates (TritAcs) and the sesquiterpene lactone taraxinic acid β-D glucopyranosyl ester (TA-G) (Huber et al. 2015). The different metabolites are produced constitutively at high concentrations. Prolonged *M. melolontha* feeding increases levels of TA-G by approximately 25%, while PIE and TriAcs are not inducible (Huber et al. 2016a). Our recent work demonstrates that TA-G repels *M. melolontha* larvae and is associated with increased plant performance (Bont et al. 2017; Huber et al. 2016b). Furthermore, *M. melolontha* pressure in the field is associated with a heritable increase in TA-G production (Huber et al. 2016a). The role of TriAcs and PIEs has not been fully resolved. While no clear correlation between PIE abundance and *M. melolontha* performance and damage was found, Di-PIEs were found to be under positive selection by leaf herbivores, indicating that they may also play a role in plant-herbivore interactions (Agrawal et al. 2018).

In this study, we asked the following questions: 1) Do root latex secondary metabolites show seasonal variations in *T. officinale*? 2) If so, can these changes be explained by fluctuating abiotic conditions such as humidity and temperature? 3) Are the changes synchronized with the probability of *M. melolontha* attack? 4) Do these changes in defense chemistry explain changes in plant attractiveness to *M. melolontha*? By combining field and growth chamber experiments, our study provides evidence that fluctuations in abiotic factors such as temperature lead to co-variation in the production of defensive metabolites and expected root herbivore attack, but also shows that other temperature-dependent factors modulate the attractiveness of *T. officinale* plants to *M. melolontha*.

## Materials and Methods

### Seasonal Variation of Secondary Metabolites in Root Latex

To investigate seasonal variation of root latex chemistry, we monitored the latex composition of a natural *T. officinale* population growing in a lawn at the Botanical Garden, University of Bern, Switzerland (46.95 °N, 7.43 °E, 520 m above sea level) over a period of 1 year. Using this natural population allowed us to randomize plant age and thereby assess seasonal fluctuations in latex chemistry independently of age. However, as dandelions show pronounced seasonality with a major flowering period from March to April (Stewart-Wade et al. 2002), ontogenetic effects cannot be fully excluded. We focused our analyses on the concentration of secondary metabolites in the latex, which have been shown to be associated with *M. melolontha* performance and preference in this system (Huber et al. 2016a, b; Bont et al. 2017). The latex was collected at monthly intervals from October 2014 to September 2015. At the beginning of each month, we randomly selected 9–12 plants, which were separated by 2–3 m from each other. Plants of approximately the same size were selected. In central Europe, field sites are typically composed of multiple genotypes, and populations in Switzerland often include both diploid outcrossers and triploid apomicts (Verduijn et al. 2004). For each plant, the roots were unearthed using a shovel. The excavated plant was mildly shaken and rinsed in water to check for root herbivore damage. Furthermore, the soil surrounding the excavated plants was searched for insect root herbivores. No *M. melolontha* larvae or other root feeding insects were found, and the excavated plant roots did not show any signs of root herbivore damage. To collect latex, the main roots were cut 0.5 cm below the tiller. The latex exuding within 10 s was collected immediately using a pre-weighted pipette tip, placed into a pre-weighted 2 ml Eppendorf tube and flash frozen in liquid nitrogen. To determine the latex mass, the tip and tube were reweighted for each sample. All samples were then stored at −80 °C until chemical analysis. To avoid diurnal fluctuations, all samples were collected between 9:00 and 10:00 AM.

### Correlation Between Secondary Metabolites and Environmental Factors

To examine the relationship between latex secondary metabolites and environmental factors, we obtained meteorological data from a nearby weather station (http://www.agrometeo.ch/de/meteorology/datas, station Noflen) during the course of a year. The station was selected based on its closely matching altitude (630 m above sea level) and its proximity to the sample site (18 km). The raw data extracted from this station included mean monthly temperature (5 cm above the ground), average monthly relative humidity and monthly precipitation. To examine the relationship between latex secondary metabolites and the damage probability by *M. melolontha* attack in nature, we used information provided by the Agricultural Education and Career Counceling Center in Landquart (Grisons, Switzerland), which has been monitoring *M. melolontha* in Switzerland for several decades. As detailed by Spinatsch (2010), *M. melolontha* typically overwinters in deeper soil layers and then gradually moves up in spring to feed on grassland plants from April to November. For plants such as *T. officinale*, which places most of its roots in the top soil, this results in a low potential damage intensity from December to March, medium probability in April, May and November and high damage probability from June–October. Accordingly, potential damage intensity (low, medium, high) was added as a factor into the analysis.

### Impact of Temperature on Secondary Metabolites in Root Latex

Seasonal fluctuations in latex secondary metabolites were strongly associated with annual changes in temperature (see results). To further investigate the influence of temperature on latex chemistry, we analyzed the latex of plants growing in climate-controlled growth chambers at different temperatures. *T. officinale* seeds were collected from 20 randomly selected plants from the same field site in May 2016. Seeds were germinated and maintained in the growth chamber with constant 50–65% relative humidity, a photoperiod of 16:8 h (light:dark), a light intensity of approximately 250 μmol*m^−2^*s^−1^ and a temperature cycle of 22:16 °C (day:night). After 2 weeks, the seedlings were individually transplanted into plastic pots (5 × 5 × 5 cm) filled with a homogenized mixture of 2/3 seedling substrate (Klasmann-Deilmann, Switzerland) and 1/3 landerde (Ricoter, Switzerland). Two weeks later, 120 plants were randomly distributed between two growth chambers with same relative humidity, photoperiod and light intensity. One growth chamber was programmed to 26 °C at day and 20 °C at night [high temperature treatment, corresponding to average temperatures in July (23.3 °C)], while another chamber was set to 18 °C at day and 12 °C at night [low temperature treatment, corresponding to average temperatures in May (14.7 °C)]. To avoid the possible difference of two chambers, plants were exchanged weekly between the two chambers, and the chambers were re-programmed each time. To eliminate the possible effects of environmental heterogeneity within the chambers, the position and direction of the pots were randomly re-arranged weekly. One month after the beginning of the temperature regimes, 30 plants from each chamber were randomly selected for latex collection and analysis.

### Indirect Impact of Temperature on Larval Preference

To determine whether plants with temperature-related differences in latex secondary metabolite concentrations vary in their defense against herbivory, we evaluated the attractiveness of the plants for *M. melolontha.* We did this by conducting a dual choice experiment with the remaining 30 plants growing in the two temperature regimes (low and high temperature), using the preference of *M. melolontha* as proxy for plant resistance. The bioassay was conducted under intermediate temperature conditions relative to the two temperature treatments (22:16 °C; day:night). Two plants (one per temperature treatment) were transplanted together into opposite sides of soil-filled rectangular arenas (20 cm length, 6 cm width, 5 cm height). The two plants were placed 10 cm apart from each other. The preference of *M. melolontha* for the plants from the different temperature treatments was assessed using a tag-and-trace system developed by Bont et al. (2017), which allows the non-invasive detecting of larvae moving freely in the soil. Third instar larvae collected from meadows in Urmein, Switzerland (46.68 ° N, 9.24 ° E) were used for the experiment. Briefly, the larvae were tagged with a thin copper wire (0.5 mm). The wire endings were twisted together to form a small antenna (0.5 cm length). The tagged larvae were starved for 1 day and then placed individually into the middle of arenas. The larvae could then move freely and feed on both plants. We monitored larval position on a centimeter scale using a commercial metal detector (Bullseye TRX Pinpointer, White’s Electronics, USA) for 1 day with 9 intervals, including 1, 2, 3, 4, 6, 8, 10, 12 and 24 h. During the bioassay, the position of the arenas was re-arranged after each measurement. After the last bioassay (24 h), the root systems were immediately harvested, and plants were scored as damaged or intact. Damage was determined by screening the root system for characteristic *M. melolontha* bite-marks. In 84% of the cases, only one of the two test plans showed signs of damage.

### Impact of TA-G on Temperature-Dependent Larval Preference

To specifically test for the role of TA-G in determining *M. melolontha* preference for plants grown at lower temperature, we conducted a second dual choice experiment with genetically modified TA-G deficient plants growing at low and high temperature in climate-controlled growth chambers. We used *T. officinale* plants in the background A34, which is a triploid line that was originally created by crossing diploid pollen of a triploid apomict from the Netherlands with a diploid mother from France (Verhoeven et al. 2010). F2 plants of a transgenic line (RNAi-1), that accumulates only little TA-G in the latex due to silenced expression of the Germacrene A synthase *ToGAS1* gene were used together with F2 plants of a transgenic control line (RNAi-15), which was transformed in an identical manner but does not exhibit *ToGAS1* silencing. The transgenic lines were characterized and described previously (Huber et al. 2016a). Plants were germinated and grown in the climate-controlled growth chambers as described above, with identical conditions and handling, until they were 8 weeks old. The dual choice experiment was conducted as described above. Larval preference for plants grown under high and low temperature was evaluated independently for TA-G deficient and TA-G producing lines. Thirty-one replicates for each choice situation were carried out at the same time. The positions of the larvae were recorded 2, 4, 6, 8, and 24 h after the start of the experiment.

### Additional Phenotypic Characterizations

To test whether the transgenic lines respond similarly to changes in temperature and to characterize additional temperature-induced phenotypic differences that may explain *M. melolontha* preference, we collected latex of 20 plants per line, washed the root system under tap water and determined fresh biomass of shoot and roots separately. Plants from the natural population collected in May 2016 were included as positive controls. Taproot latex was obtained by pipetting 2 μl of the exuding latex into 200 μl 100% methanol (HPLC-grade). Samples were stored at −80 °C until extraction. From the 20 replicates, we quantified TA-G, Di-PIEs and Tri-PIEs for 13–19 replicates per line, because not all plants produced enough latex for quantification. Total protein was determined for the side roots of 11–13 out of the 20 replicates. Roots were flash frozen in liquid nitrogen, ground to a fine powder and protein content was quantified according to Bradford (Bradford 1976).

### Analyses of Secondary Metabolites

Extraction, analysis and quantification of secondary metabolites in the root latex were performed as described by Bont et al. (2017) and Huber et al. (2015) with a few slight modifications. Briefly, 1 ml 100% methanol (HPLC-grade) was added to the tubes with latex collected with pre-weighted pipette tips, and all tubes were vortexed 10 min at room temperature and centrifuged for 20 min at 13000 rpm. The supernatant was transferred into a 2 ml glass tube. Latex MeOH extracts were immediately analyzed by an UPLC-PDA-MS (Waters). Auto-injected samples of 2.5 μl volume were separated on an Acquity BEH-C18 column (100 × 2.1 mm, 1.7 μm particles, Waters), with the column temperature being 55 °C. The flow rate was 0.4 ml min^−1^. The mobile phase A was 99.9% H_2_O and 0.1% formic acid, and the mobile phase B was 99.9% acetonitrile and 0.1% formic acid. The gradient run was as follows: 0 min: 95% A; 5.5 min 90% A; 13 min 80% A; 14 min 65% A; 14.1 min 5% A, 15.6 min 95% A. The concentration of TA-G was determined by measuring peak areas at 245 nm, calculated using loganin as the external standard (Sigma-Aldrich, Switzerland), and expressed as μg mg^−1^ latex fresh mass for the latex collected with pre-weighted pipette tips, whereas for the latex collected by pipetting relative concentrations were calculated. Relative concentrations of PIEs were determined by measuring peak areas at 275 nm and expressed as peak area mg^−1^ latex fresh mass for the latex collected with pre-weighted pipette tips and as relative concentrations for the latex collected by pipetting. Di- and Tri-PIEs were calculated separately.

### Data Analysis

To test whether the concentration of metabolite classes in the root latex varies over the year, the concentrations of individual metabolites were summed to obtain total Di-PIEs, Tri-PIEs and TA-G and were analyzed using One-way analysis of variance (ANOVA) with month as a fixed factor. Multiple comparisons were carried out using least square mean post-hoc tests (LSM) and *p*-values were corrected using the False Discovery Rate (FDR) method (Benjamini and Hochberg 1995). To examine whether the overall metabolite profile in the root latex varies between different months, all detected PIEs as well as TA-G were subjected to a redundancy analysis (RDA) after centering and scaling. Monte Carlo test (default setting of 999 permutations) was used to test for significant differences between months. To examine the relationships between metabolite classes and environmental factors, pairwise correlation tests were performed, and *p*-values were corrected for multiple testing using the FDR method. Pearson correlation was used for all environmental factors except damage potential by *M. melolontha* larvae, which for which Spearman correlations were employed. RDA was also used to examine the possible correlations between overall metabolite profiles and environmental factors (damage potential by *M. melolontha* larvae considered as a qualitative variable). To examine the impact of temperature on the different metabolite classes in the root latex of plants from the natural population, total Di-PIEs, Tri-PIEs and TA-G were analyzed using independent sample t-tests (high vs. low temperature). To test for an effect of the temperature regime on the overall metabolite profiles in the root latex, RDA and associated Monte Carlo test was used as above. To determine the preference of *M. melolontha* larvae between the different temperatures treated plants from the natural population, the rectangular arenas (20 cm length) were divided into 3 zones. The central zone of 4 cm length was defined as neutral area, and the side zones of 8 cm length were defined as preference areas. Because the number of larvae in the central area did not differ between choice situations, they were excluded from further analyses. Larval preferences at individual time points were compared using binomial tests. To test whether larval preference varies with time, the number of larvae found on low or high temperature plants were analyzed using a Wald test applied to a generalized linear mixed model (GLMM, binomial distribution) with time as a fixed factor and arena as a random factor. To test whether the larval choice varies with time, the number of larvae found on both plants was analyzed using a Wald test as describe above. Differences in plant damage were tested using a binomial test. To examine temperature-induced phenotypic differences of transgenic plants and plants from the natural population, TA-G, Di-PIEs, Tri-PIEs, root protein content, shoot and root biomass were compared between plants growing at low temperature and plants growing at high temperatures separately for each transgenic line and for the plants from the natural population using independent sample t-tests. To compare the preference of *M. melolontha* larvae between the different temperatures treated transgenic plants that either contain TA-G in normal quantities (RNAi-15) or are impaired in TA-G production (RNAi-1), we established a linear mixed model. We calculated the probability of choice for each test plant for each larva by determining the percentage of detections in the corresponding side. ‘Probability of choice’ was then used as response variable, whereas ‘Temperature’ (if the plant was growing at high or low temperature), ‘Genotype’ (either RNAi-15 or RNAi-1) and the interaction of both were the explanatory variables in the model. As the probability of choice is calculated from repeated measurements of the same larvae, ‘(1|larva)’ was added as random factor. The model was tested using Wald test. All data were analyzed using R 3.2.0 (R Foundation for Statistical Computing, Vienna, Austria) with ‘car’, ‘lme4’, ‘lsmeans’, ‘vegan’ and ‘RVAideMemoire’ packages (Bates et al. 2015; Fox and Weisberg 2011; Hervé 2018; Lenth 2016; Oksanen et al. 2016).

## Results

### Seasonal Patterns in Root Secondary Metabolite Accumulation

**Fig. 1:**
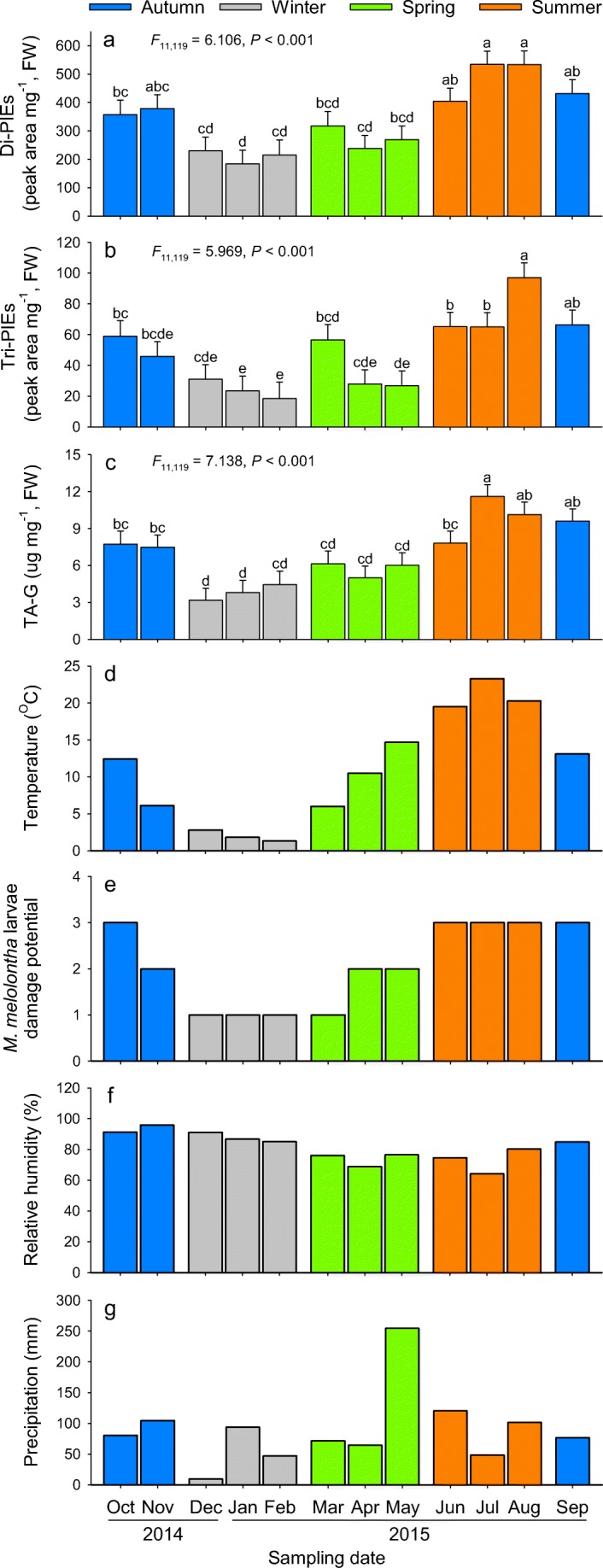
Seasonal variation in *Taraxacum officinale* root secondary metabolites and environmental parameters. Total latex concentrations are shown for Di-PIEs **a**, Tri-PIEs **b** and TA-G **c** from plants from a naturally growing field population without any *Melolontha melolontha* larvae (mean ± 1 SE, *n* = 9 to 12 plants per month). Environmental parameters include mean monthly temperature **d**, relative damage potential by *M. melolontha* larvae (1 = low, 2 = medium, 3 = high) **e**, average monthly relative humidity **f** and monthly precipitation **g**. Gray, green, orange and blue bars represent winter (December, January and February), spring (March, April and May), summer (June, July and August) and autumn (September, October and November), respectively. Differences between months were determined by One-Way ANOVAs followed by post hoc multiple comparisons (LSM) and *P*-values corrections (FDR)

The monitoring of the latex secondary metabolites in the natural population shows that the concentrations of total Di-PIEs, Tri-PIEs and TA-G changed significantly throughout the season (Di-PIEs, *F*_11,119_ = 6.106, *P* < 0.001; Tri-PIEs, *F*_11,119_ = 5.969, *P* < 0.001; TA-G, *F*_11,119_ = 7.138, *P* < 0.001). Generally, concentrations were low from December to February, increased steadily from March onwards, peaked in August and declined from September to December (Fig. 1a–c). The only deviation from this pattern was a transient drop between March and April, which marked the start of the flowering period (Fig. 1a–c). Across the year, TA-G concentrations were strongly correlated with total Di-PIEs (*r* = 0.865, *P* < 0.001, Fig. S1a) and Tri-PIEs (*r* = 0.617, *P* < 0.001, Fig. S1b). In addition, total Di-PIEs and Tri-PIEs were also positively correlated with each other (*r* = 0.655, *P* < 0.001, Fig. S1c). Consistent with the overall patterns for the different metabolite classes, metabolite profiles including all individual Di- and Tri-PIEs and TA-G were also significantly affected by the season (Month, *F*_11,119_ = 3.909, *P* < 0.001, Fig. S2a). No significant effect of the season on the mass of collected latex was found (*F*_11,119_ = 1.039, *P* = 0.417, Fig. S3).

### Correlations Between Environmental Factors and Secondary Metabolite Accumulation

**Fig. 2:**
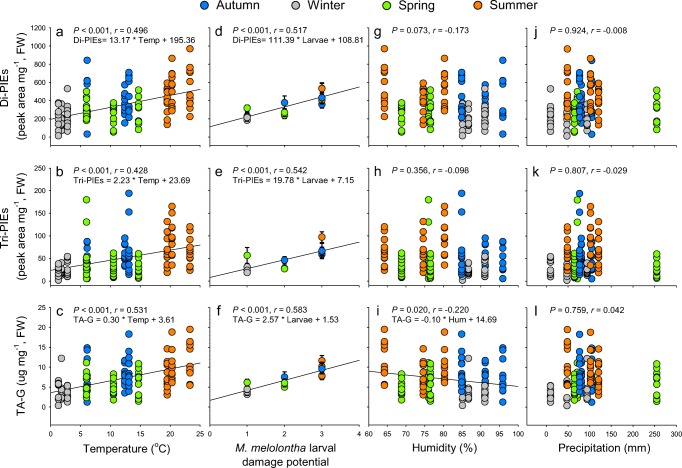
Correlations between environmental parameters and *Taraxacum officinale* root secondary metabolites. Solid lines represent linear correlations. Points in **a**–**c** (temperature), **g**–**i** (humidity) and **j**–**l** (precipitation) represent individual plants (Pearson correlation, n = 9 to 12 plants per month, 131 plants in total). Points with error bars in **d**–**f** (larval damage potential) indicate mean of each month (Spearman correlation). Colors represent the different seasons as described in Fig. 1. *P* and *r* values from corresponding correlations are shown

Visual inspection of the seasonal fluctuation curves indicated co-variation of all secondary metabolite classes with temperature and *M. melolontha* damage probability, with the exception of the drop between March and April, (when plants started flowering) which was not tracked by the two environmental factors (Fig. 1d, e). Humidity and precipitation did not exhibit any clear seasonal patterns (Fig. 1f, g). Correlation analysis revealed that concentrations of total Di-PIEs, Tri-PIEs and TA-G were positively correlated with temperature (Di-PIEs, *r* = 0.496, *P* < 0.001; Tri-PIEs, *r* = 0.428, *P* < 0.001; TA-G, *r* = 0.531, *P* < 0.001; Fig. 2a–c) and larval damage potential (Di-PIEs, *r* = 0.517, *P* < 0.001; Tri-PIEs, *r* = 0.542, *P* < 0.001; TA-G, *r* = 0.583, *P* < 0.001; Fig. 2d–f). Total Di-PIEs and Tri-PIEs were not correlated with humidity (Di-PIEs, *r* = −0.173, *P* = 0.073; Tri-PIEs, *r* = −0.098, *P* = 0.356; Fig. 2g, h), while TA-G was negatively correlated with humidity (*r* = −0.220, *P* = 0.020, Fig. 2i). There was no correlation between metabolite classes and precipitation (Di-PIEs, *r* = −0.008, *P* = 0.924; Tri-PIEs, *r* = −0.029, *P* = 0.807; TA-G, *r* = 0.042, *P* = 0.759; Fig. 2j–l). Similar relationships were also found for total metabolite profiles (larval damage potential, *r*^2^ = 0.152, *P* < 0.001; temperature, *r*^2^ = 0.288, *P* < 0.001; humidity, *r*^2^ = 0.030, *P* = 0.132; precipitation, *r*^2^ = 0.012, *P* = 0.471; Fig. S2b, c).

### Effect of Temperature on Root Secondary Metabolites

**Fig. 3:**
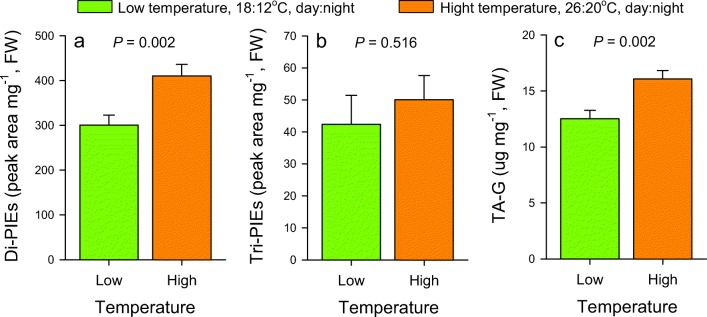
Impact of temperature on *Taraxacum officinale* root secondary metabolites. Total Di-PIEs **a**, Tri-PIEs **b** and TA-G **c** from plants growing at low (green: 18:12 °C, day:night, corresponding to May) and high (orange: 26:20 °C, day:night, corresponding to July) temperatures for 1 month are shown (mean ± 1 SE, *n* = 30 plants per treatment). Differences between treatments were determined by independent sample *t*-tests

In the growth chamber experiment with plants from the natural population, temperature significantly affected the concentrations of total Di-PIEs (*t* = 3.178, *P* = 0.002) and TA-G (*t* = 3.322, *P* = 0.002), but had no impact on Tri-PIEs (*t* = 0.654, *P* = 0.516). Plants growing at higher temperature had higher concentrations of Di-PIEs and TA-G than plants in the low temperature chamber (Fig. 3). Similar patterns were found for total metabolite profiles (*F*_1,58_ = 7.580, *P* < 0.001, Fig. S4).

### Effect of Temperature on Larval Preference

**Fig. 4:**
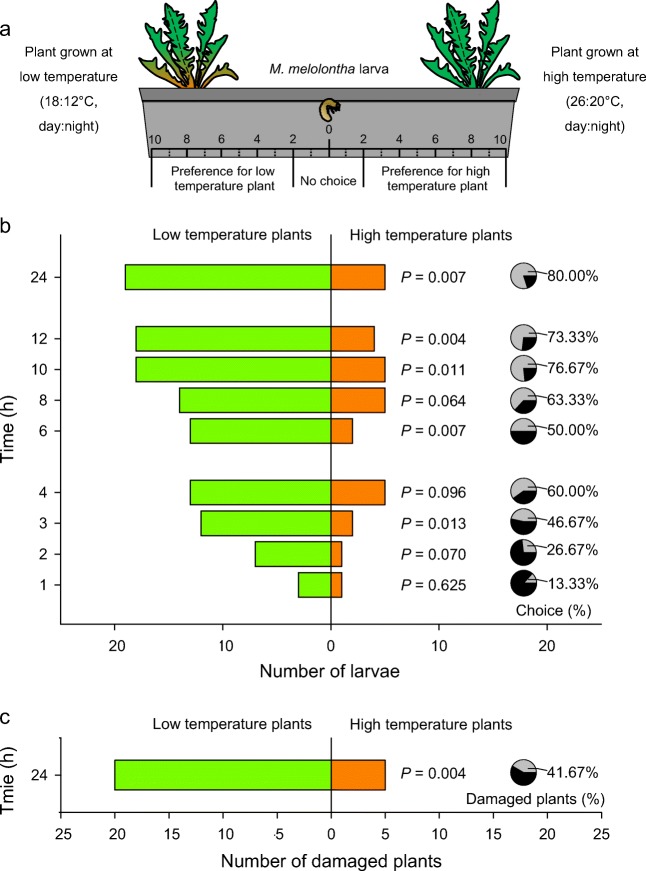
Impact of plant growth temperature on *Taraxacum officinale* root resistance. **a** Experimental set-up. Two plants, (one per temperature treatment as described in Fig. 3) were transplanted together into opposite sides of soil-filled rectangular arenas. The bioassay was then conducted under intermediate temperature conditions (22:16 °C; day:night) for 1 day. **b** Number of *Melolontha melolontha* larvae in the vicinity of *T. officinale* plants which were previously grown at low (green, left) or high (orange, right) temperature. Pie charts on the right show the proportion of larvae that enter the preference area at each time point. **c** Number of damaged plants after 24 h. Differences in larval preferences at individual time points and in plant damage at the end of experiment were tested using binomial tests

In the bioassay with plants from the natural population (Fig. 4a), *M. melolontha* larvae showed a strong preference for plants growing at low temperature at 3 h (*P* = 0.013), 6 h (*P* = 0.007), 10 h (*P* = 0.011), 12 h (*P* = 0.004), and 24 h (*P* = 0.007) (Fig. 4b). The percentage of larvae making a choice significantly increased with time (*X*^2^ = 29.495, *P* < 0.001), from 13% at 1 h to 80% at 24 h (Fig. 4b). However, larval preference did not change over time (*X*^2^ = 1.318, *P* = 0.251, Fig. 4b). At the end of the bioassay (24 h), 42% of plants showed visible signs of damage. Significantly more plants growing at low temperature were damaged than plants growing at high temperature (*P* = 0.004, Fig. 4c).

### Impact of Temperature on Root Secondary Metabolites, Protein Contents and Plant Biomass

**Fig. 5:**
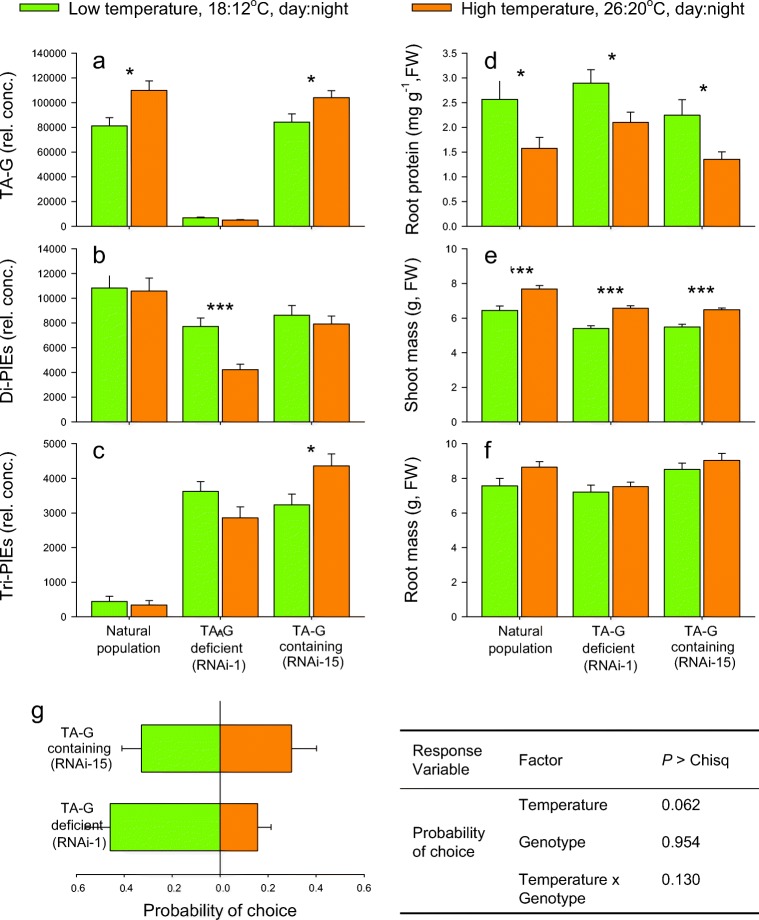
Impact of growth temperature on phenotype and attractiveness to *Melolontha melolontha* of TA-G producing and TA-G deficient plants. TA-G **a**, Di-PIEs **b**, Tri-PIEs **c**, root protein content **d**, shoot **e** and root biomass **f** from plants from natural population, TA-G deficient (RNAi-1) and TA-G producing (RNAi-15) plants growing at low (18:12 °C, day night, corresponding to May) and high (26:20 °C, day:night, corresponding to July) temperatures for 1 month are shown (mean ± 1 SE, *n* = 11–20 plants). Differences between treatments were determined by independent sample *t*-tests. **g** Dual choice experiment for testing the preference of *M. melolontha* between transgenic plants from low and high temperatures, whereas plants were either TA-G producing (RNAi-15) or TA-G deficient (RNAi-1). Overall probability of choice (± 1 SE) for each test plant is shown, which is calculated from five measurements of the larvae’s position over 24 h. The role of TA-G for the larvae’s preference was analyzed in a mixed effect model and *P-*values for each explanatory factor are noted

As seen before, TA-G levels increased in plants grown at higher temperatures in the natural population (*t* = 2.583, *P* = 0.014) as well as in plants from the transgenic control line RNAi 15 (*t* = 2.306, *P* = 0.028) (Fig. 5a). By contrast, TA-G levels were reduced by more than 90% in the RNAi-1 plants, and temperature had no effect on the residual TA-G (*t* = 1.617, *P* = 0.117, Fig. 5a). The concentration of Di-PIEs was decreased in RNAi-1 plants when growing at high temperatures (*t* = 4.255, *P* < 0.001), whereas temperature treatment had no effect on Di-PIEs production of plants from the natural population (*t* = 0.167, *P* = 0.869) and RNAi-15 plants (*t* = 0.701, *P* = 0.488) (Fig. 5b). High temperature treatment increased Tri-PIEs in RNAi-15 plants (*t* = 2.437, *P* = 0.021) but had no effect on Tri-PIEs in plants from the natural population (*t* = 0.510, *P* = 0.613) and in RNAi-1 plants (*t* = 1.813, *P* = 0.082) (Fig. 5c). All plants growing at high temperatures had lower protein content in their side roots (natural population, *t* = 2.189, *P* = 0.043; RNAi-1, *t* = 2.32, *P* = 0.030; RNAi-15, *t* = 2.542, *P* = 0.022; Fig. 5d). Higher temperatures increased shoot biomass (natural population, *t* = 3.804, *P* < 0.001; RNAi-1, *t* = 5.587, *P* < 0.001; RNAi-15, *t* = 5.216, *P* < 0.001; Fig. 5e). Root biomass was not significantly affected by temperature for any of the genotypes (natural population, *t* = 1.964, *P* = 0.058; RNAi-1, *t* = 0.636, *P* = 0.529; RNAi-15, *t* = 1.133, *P* = 0.294; Fig. 5f). Thus, summer temperatures consistently increase TA-G levels, while the impact on PIEs seems to be more context dependent and variable. Furthermore, changes in temperature are accompanied by modifications in plant primary metabolism.

### Impact of TA-G on Temperature-Dependent Larval Preference

The bioassay with transgenic plants growing at low and high temperature confirmed that the larvae prefer plants growing at low temperature (Fig. 5g), albeit with only marginal significance for this genetic background (Temperature, *P* = 0.062). Whether the plants were TA-G deficient (RNAi-1) or TA-G producing (RNAi-15) had no effect on larval choice (Genotype, *P* = 0.954; Temperature x Genotype, *P* = 0.130; Fig. 5g). Thus, the lower TA-G levels are not sufficient to explain the preference of *M. melolontha* larvae for plants grown at low temperature (Fig. 5g).

## Discussion

In order to maximize their chance of survival and reproduction, plants may evolve to deploy their defenses when they are most needed, as proposed by the Optimal Defense Theory (McKey 1974, 1979). Fluctuating environmental conditions can be a challenge for optimal defense deployment, as they may affect the expression of defense traits (Coley et al. 1985). However, as herbivore attack may also vary with environmental conditions, these conditions may also be used by plants to synchronize defense deployment with anticipated herbivore attack. Our work indicates a role for temperature to synchronize the deployment of secondary metabolites, including a well-documented repellent, with the probability of attack by a major root feeding natural enemy over the course of the year. At the same time, however, phenotypic changes other than the accumulation of secondary metabolites contribute to reduce the attractiveness of plants growing at higher temperatures. Below, we discuss these findings in more detail.

Seasonal variation in secondary metabolites has been documented repeatedly in above ground plant tissues (Bowers et al. 1992; Carvalho et al. 2014; Feeny 1970; Hare 2010; Liimatainen et al. 2012; Riipi et al. 2002). Several recent studies also demonstrate seasonal variability in secondary metabolites in below ground plant tissue (Ciska et al. 2017; Robakowski et al. 2016). Our results further support the notion that seasonal variation in defenses is common in below ground plant tissues. The biosynthesis of secondary metabolites can be modified by a wide range of environmental factors, such as temperature, precipitation and photoperiod (Akula and Ravishankar 2011; Gutbrodt et al. 2011; Pellissier et al. 2014). We found that Di-PIEs, Tri-PIEs and TA-G were positively correlated with temperature changes throughout the season, suggesting that temperature fluctuations may contribute to the seasonal patterns observed in the field. Our growth chamber experiment supports this hypothesis for TA-G, but not Tri-PIEs and only partially for Di-PIEs. Temperature profoundly affects many primary processes in plants, including photosynthesis and biochemical conversion processes (Berry and Björkman 1980; McClung and Davis 2010). Plants are therefore highly sensitive to temperature fluctuations, and temperature cycles can entrain their circadian and circannual clocks (James et al. 2012; McWatters and Devlin 2011). *T. officinale* may therefore use temperature fluctuations across the season to establish its biological circannual clock and specifically regulate its defenses. Alternatively, defenses may also be regulated directly by temperature-dependent metabolic changes.

Interestingly, Di-PIEs Tri-PIEs and TA-G transiently decreased in April, which coincided with the onset of flowering, but not environmental factors or risk of herbivore attack. Plant ontogeny can affect resource allocation to defense traits (Barton and Koricheva 2010; Boege and Marquis 2005). Several studies demonstrate that the production of defenses is reduced during the transition from vegetative to generative growth (Diezel et al. 2011). We speculate that *T. officinale* prioritizes reproduction over defense, resulting in resource re-allocation that constrains the production of secondary metabolites. Further work is required to disentangle the relative contribution of environmental factors and plant ontogeny to seasonal variation (Barton and Boege 2017) and to understand how resource allocation shapes defensive chemistry in this plant system.

Secondary metabolites play a crucial role in plant defense against herbivores through toxic and feeding deterrent effects as well as the attraction of natural enemies of herbivores (Mithöfer and Boland 2012; War et al. 2012). Since defenses can be costly, plants should maintain high defense levels only when they are attacked by herbivores (McKey 1974). Our recent work shows that TA-G is a potent defense against *M. melolontha,* which benefits the plant in the presence of the root feeder, but reduces seed production in its absence (Bont et al. 2017; Huber et al. 2016a, b). In addition, many studies have shown that temperature is a good indicator of insect herbivore attack, since development, survival and appearance of herbivores in the field are generally modulated by temperature (Bale et al. 2002; Ratte 1985). *M. melolontha* is no exception to this rule (Spinatsch 2010). Thus, *T. officinale* may use seasonal temperature variation to synchronize defense deployment with expected herbivore attack intensity to maximize its fitness. This pattern represents a possible strategy that perennial plants can use to time the deployment of herbivore defenses during the course of the year. Interestingly, TA-G deployment is synchronized with the activity of the major root herbivore of *T. officinale*, *M. melolontha*, but not with wireworms, who are less abundant on *T. officinale* (Huber et al. 2016a) and are most active in Spring (Jung et al. 2012). Thus, temperature may be a specific Zeitgeber for the most damaging root herbivore that dandelion faces in Switzerland and Germany. In this context, it is important to note, however, that *M. melolontha* preference for plants grown at low temperature did not require intact TA-G biosynthesis. Thus, other temperature dependent factors such as primary metabolites and protein levels, all of which are known to influence the behavior of root herbivores (Erb et al. 2013) also have the potential to shape *M. melolontha* feeding. We speculate that, all else being equal, dandelions in natural populations may benefit from accumulating TA-G during the summer months by lowering the chance of being attacked relative to neighbors that do not employ this type of synchronization. Testing this hypothesis would require further experiments with TA-G deficient, and, ideally, TA-G desynchronized plants in their natural environments.

In conclusion, our findings suggest that an integrated view of seasonal variation and temperature-dependent changes is essential to understand how plants synchronize their metabolism with specific seasonal growth requirements and the need to deploy defenses at the right time.

## Electronic Supplementary Material

ESM 1 (PDF 229 kb)
