# Supplementary material for: Impact of Seasonal and Temperature-Dependent Variation in Root Defense Metabolites on Herbivore Preference in Taraxacum officinale
Source: J Chem Ecol. 2019 Dec 12;46(1):63–75. doi: 10.1007/s10886-019-01126-9 (PMC6954900; doi:10.1007/s10886-019-01126-9)
Supplement: Supplementary file 1 — (PDF 229 kb) [file 10886_2019_1126_MOESM1_ESM.pdf]

Fig. S1

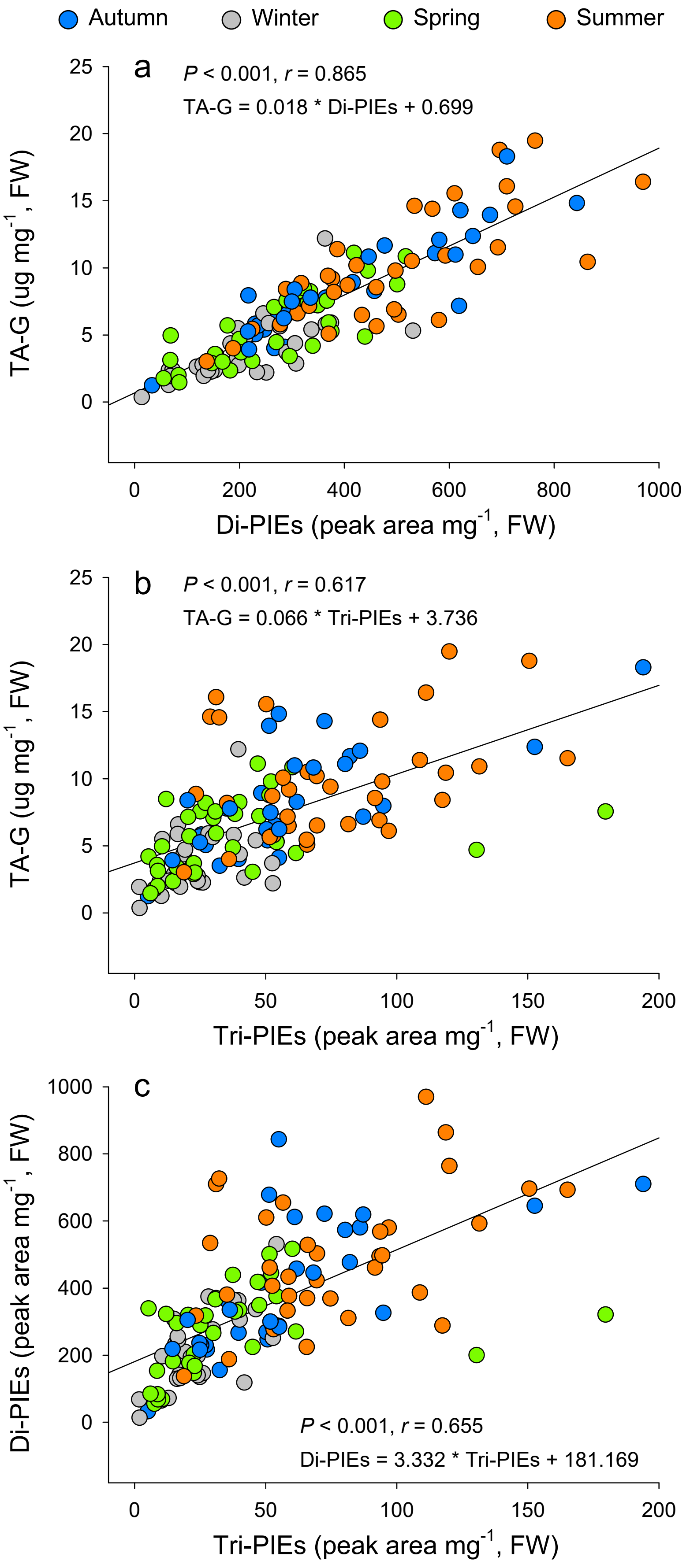

Autumn
Winter
Spring
Summer
a
P < 0.001, r = 0.865
TA-G = 0.018 * Di-PIEs + 0.699
TA-G (ug mg-1, FW)
Di-PIEs (peak area mg-1, FW)
b
P < 0.001, r = 0.617
TA-G = 0.066 * Tri-PIEs + 3.736
TA-G (ug mg-1, FW)
Tri-PIEs (peak area mg-1, FW)
c
P < 0.001, r = 0.655
Di-PIEs = 3.332 * Tri-PIEs + 181.169
Di-PIEs (peak area mg-1, FW)
Tri-PIEs (peak area mg-1, FW)

Fig. S2

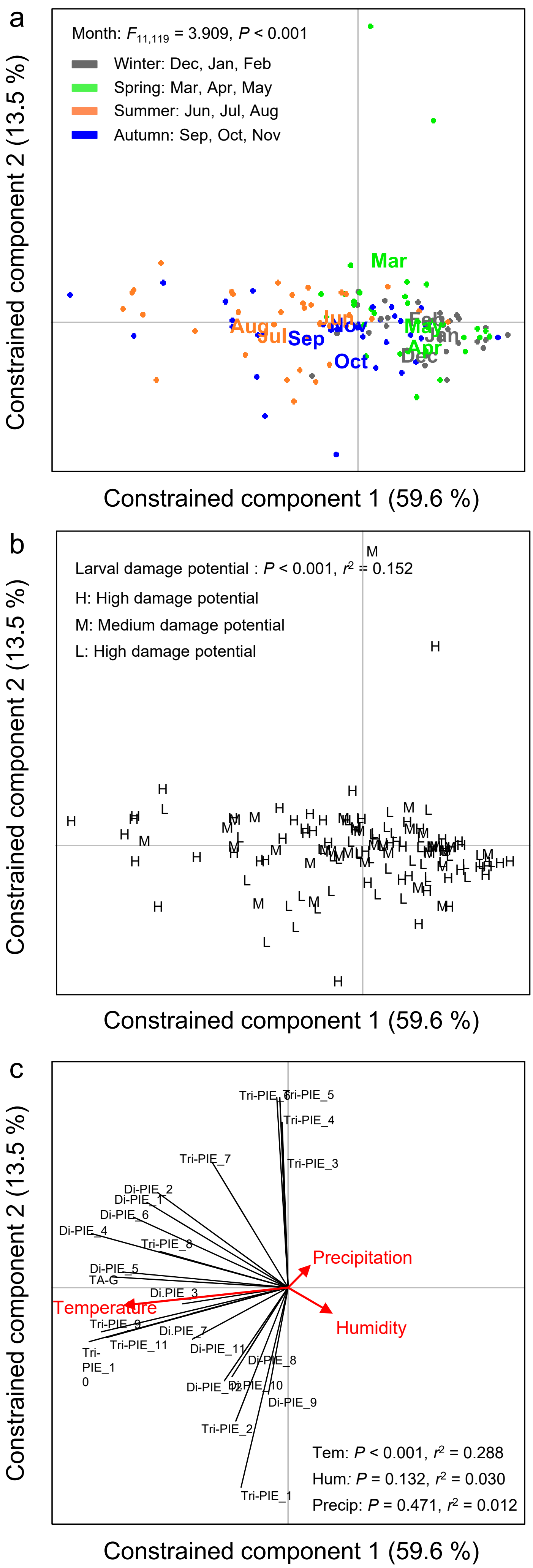
a
Month: $F_{11,119}$ = 3.909, P < 0.001
Winter: Dec, Jan, Feb
Spring: Mar, Apr, May
Summer: Jun, Jul, Aug
Autumn: Sep, Oct, Nov
Mar
Aug
Jul
Sep
Jun
Nov
Oct
Feb
May
Jan
Apr
Dec
Constrained component 1 (59.6 %)
Constrained component 2 (13.5 %)
b
Larval damage potential : P < 0.001, r^2 = 0.152
H: High damage potential
M: Medium damage potential
L: High damage potential
Constrained component 1 (59.6 %)
Constrained component 2 (13.5 %)
c
Tri-PIE_6
Tri-PIE_5
Tri-PIE_4
Tri-PIE_7
Tri-PIE_3
Di-PIE_2
Di-PIE_1
Di-PIE_6
Di-PIE_4
Tri-PIE_8
Precipitation
Di-PIE_5
TA-G
Di.PIE_3
Temperature
Humidity
Tri-PIE_9
Di.PIE_7
Tri-PIE_11
Tri-PIE_10
Di-PIE_11
Di-PIE_8
Di-PIE_12
Di-PIE_10
Di-PIE_9
Tri-PIE_2
Tri-PIE_1
Tem: P < 0.001, r^2 = 0.288
Hum: P = 0.132, r^2 = 0.030
Precip: P = 0.471, r^2 = 0.012
Constrained component 1 (59.6 %)
Constrained component 2 (13.5 %)

Fig. S3

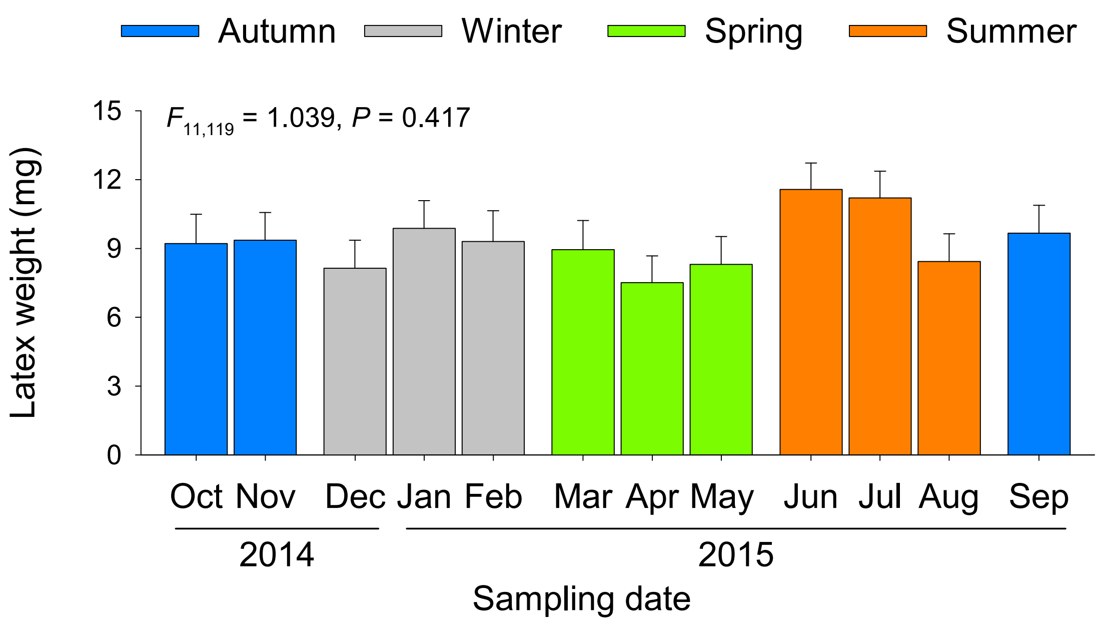
Autumn
Winter
Spring
Summer
$F_{11,119}$ = 1.039, P = 0.417
Latex weight (mg)
15
12
9
6
3
0
Oct Nov Dec Jan Feb Mar Apr May Jun Jul Aug Sep
2014
2015
Sampling date

Fig. S4

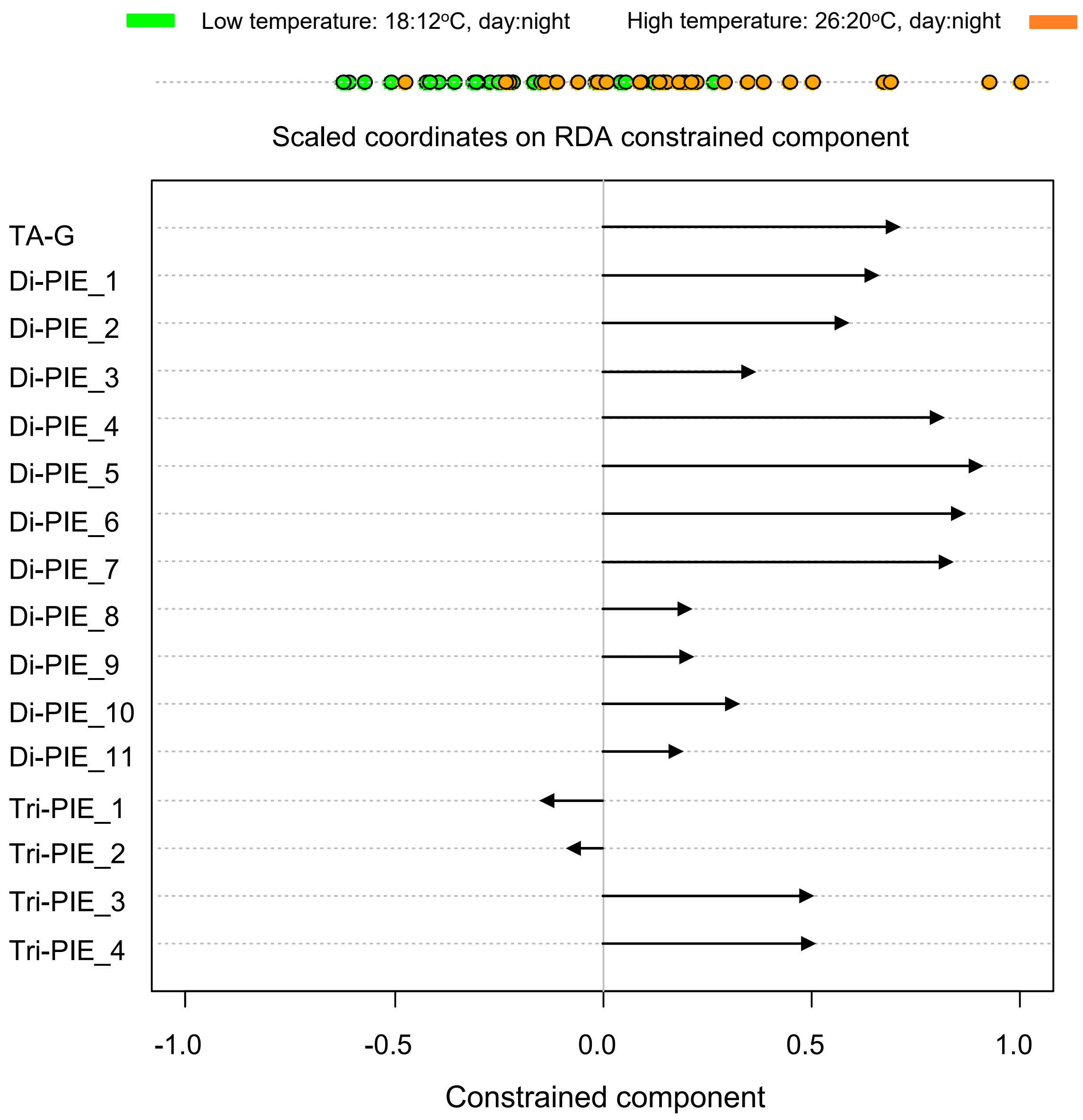
Low temperature: 18:12ºC, day:night
High temperature: 26:20ºC, day:night
Scaled coordinates on RDA constrained component
TA-G
Di-PIE_1
Di-PIE_2
Di-PIE_3
Di-PIE_4
Di-PIE_5
Di-PIE_6
Di-PIE_7
Di-PIE_8
Di-PIE_9
Di-PIE_10
Di-PIE_11
Tri-PIE_1
Tri-PIE_2
Tri-PIE_3
Tri-PIE_4
-1.0
-0.5
0.0
0.5
1.0
Constrained component
